# Therapy With Different Dose Regimens of Rituximab in Patients With Active Moderate-To-Severe Graves’ Orbitopathy

**DOI:** 10.3389/fendo.2021.790246

**Published:** 2022-01-25

**Authors:** Irene Campi, Guia Vannucchi, Ilaria Muller, Elisa Lazzaroni, Nicola Currò, Martina Dainese, Benedetta Montacchini, Danila Covelli, Claudio Guastella, Lorenzo Pignataro, Laura Fugazzola, Maura Arosio, Mario Salvi

**Affiliations:** ^1^ Department of Endocrine and Metabolic Diseases, Istituto Auxologico Italiano IRCCS, Milan, Italy; ^2^ Graves’ Orbitopathy Center, Endocrinology, Fondazione IRCCS Cà Granda, Ospedale Maggiore Policlinico Milan, Milan, Italy; ^3^ Department of Clinical Sciences and Community Health, University of Milan, Milan, Italy; ^4^ Division of Infection and Immunity, School of Medicine, Cardiff University, Cardiff, United Kingdom; ^5^ Ophthalmology, Fondazione IRCCS Cà Granda, Ospedale Maggiore Policlinico Milan, Milan, Italy; ^6^ Otolaryngology, University of Milan and Fondazione IRCCS Cà Granda, Milan, Italy; ^7^ Department of Pathophysiology and Transplantation, University of Milan, Milan, Italy

**Keywords:** Graves’ disease, Graves’ orbitopathy, Rituximab, B lymphocytes, TSH-receptor antibodies

## Abstract

**Background:**

Immunosuppressive therapy of Graves’ orbitopathy (GO) is indicated during the active phase of disease. Intravenous steroids (IVGC) are effective in about 70% of patients, although unresponsiveness or relapse are observed. In previous studies, rituximab (RTX) has been shown to be effective in inactivating moderate-to-severe GO when used early in the disease, but its optimal dosage has never been studied in randomized clinical trials. Aim of this study was to compare the efficacy and safety of different doses of RTX, based on a *post-hoc* analysis of two open label studies and one prospective trial randomized to IVGC.

**Methods:**

of 40 patients (35 women, 5 men), with active moderate-to-severe GO treated with RTX, 14 received a single dose of 100 mg (Group 1), 15 a single dose of 500 mg (Group 2) and 11 two 1000 mg doses, administered one week apart (Group 3). Thyroid function, TSH-receptor antibodies (TRAb) and peripheral CD19+ cells were measured. Primary endpoint was disease inactivation, measured as a decrease of the Clinical Activity Score (CAS) of at least two points. Secondary endpoints were improvement of proptosis, diplopia, quality of life and safety.

**Results:**

Baseline CAS decreased significantly in all groups (P<0.0001), independently of GO duration or whether patients had newly occurring or relapsing GO after IVGC. Proptosis did not significantly change. There was an inverse correlation between the Gorman score for diplopia and RTX dose (P<0.01). The appearance score of the GO-QoL improved in Group 1 (P=0.015), and the visual function score, in Group 2 (P=0.04). A reduction of serum TRAb was observed in Group 1 (P=0.002) and Group 2 (P<0.0002), but not in Group 3. CD19+ cell decreased in all groups (P<0.01), independently of the dose.

**Conclusions:**

We studied the optimal dosage of RTX in the treatment of active moderate-to-severe GO. In this analysis, we considered the efficacy of RTX in inactivating GO, in changing its natural course, its effect on disease severity and on the patients’ quality of life. Based on our clinical findings, and balancing the cost of therapy, a single 500 mg dose regimen is suggested in the majority of patients.

## Introduction

Graves’ disease (GD) is a thyroid autoimmune disorder in which anti-thyrotropin (TSH) receptor autoantibodies (TRAb) with stimulating activity induce hyperthyroidism. Graves’ orbitopathy (GO), characterized by inflammation and remodelling of orbital tissues, is the main extra-thyroidal manifestation of GD. Moderate-to-severe GO determines major facial disfigurement, psychosocial impact and, rarely progression to sight loss (1, 2). Typically, GO has a diphasic course (3) with an inflammatory and progressive phase, followed by stabilization and subsequent scarring due to tissue fibrosis. Medical treatment, based on immunosuppression, is indicated during the active phase of GO. Intravenous steroids have been shown to be effective in about 70% of patients, although unresponsiveness and disease relapse are observed in a good proportion of patients (20-30%) (4).

Rituximab (RTX) is a human/mouse chimeric monoclonal antibody that binds the CD20 antigen on the surface of B cells and induces rapid depletion of B cells in both periphery and lymphoid organs. Clinically approved indications for the use of RTX in autoimmune disease are currently rheumatoid arthritis and ANCA-related vasculitis (5). In previous studies (6), RTX has been shown to be effective in inactivating moderate-to-severe GO when used early in the disease, but may have little effect in longer duration disease (7). The clinical effect of RTX in GO is quite rapid, since disease generally inactivates within 4 – 6 weeks after the first infusion (8). RTX induces B-cell lysis and may activate anaphylatoxins and other inflammatory cytokines with subsequent recruitment of phagocytes within the orbital tissue and depletion of inflammatory cells yielding tissue fibrosis (9, 10). This may explain why, after B cells repopulation, reactivation of GO was only observed rarely.

RTX therapy is not devoid of side effects: infections may arise due to hypogammaglobulinemia, although they are also commonly associated to therapy with steroids or other immunosuppressive drugs (11). Acute infusion reactions related to the release of cytokines produced by macrophages, monocytes, lymphocytes and NK cells, can be observed in 10-30% of patients at the first infusion (12). These effects may be associated to larger doses of RTX and significant lymphocytic infiltrates in target organs (13).

The optimal dosage of RTX in GO has never been studied in randomized, dose finding clinical trials. Standard dosing in rheumatoid arthritis and other autoimmune diseases consists of two i.v. administrations of 1000 mg with a 2-week interval. This dose was also initially used in most open studies (8) and in individual case reports (10) in patients with GO. Rapid and complete B lymphocyte depletion was shown to occur already after very low dosages (25-100 mg) of i.v. RTX (9). In a prospective randomized clinical study it was shown that the efficacy of a single 500 mg RTX dose was comparable to two 1000 mg doses (7) in inactivating GO and more effective than i.v. methylprednisolone (6). Later observation that in GO total peripheral (and orbital) B cell depletion was occurring even after low doses of RTX (100 mg) (9), has allowed us to reduce the therapeutic dose to a single infusion of 100 mg RTX (14).

Aim of the present study was to compare the efficacy and safety of different doses of RTX, employed prospectively in two open label studies (14, 15) and in one trial in which RTX was randomized to i.v. steroid therapy (6), in active GO.

## Patients and Methods

### Patients

Forty patients, 35 women and 5 men, 32-81 years of age (mean ± SE 58.7 ± 2.7 yr), with active moderate to severe GO were grouped based on the RTX dose that they received (**Table 1**). Of these patients, 14 were treated with a single dose of 100 mg (Group 1), 15 with a single dose of 500 mg (Group 2) and 11 with two 1000 mg doses of RTX, administered one week apart (Group 3). Of the 40 patients included in the study, 34 had GD, 11 in group 1, 12 in group 2 and 11 in group 3, five had Hashimoto’s thyroiditis, of whom three in Group 1 and two in Group 2, and one had euthyroid GO (Group 2). Thirty of 34 GD patients were euthyroid or subclinical hypothyroid because slightly overtreated with methimazole and four slightly hyperthyroid at the time of treatment. Nineteen patients were smokers, not significantly distributed in the three groups (**Table 1**). All patients had mean GO duration of 11 ± 3 months. Nineteen patients were previously treated with corticosteroid therapy, nine in Group 1, six in Group 2 and four in Group 3. Glucocorticoids were discontinued at least three months before RTX administration. Patients were followed-up to 76-weeks follow-up to assess the need of rehabilitative surgery.

**Table 1:** Baseline clinical characteristics of patients with moderate-to-severe GO treated with different doses of RTX.

Group (dose of RTX)	GROUP 1 (100 mg)	GROUP 2 (500 mg)	GROUP 3 (1000 mg x2)	*P*
Number of patients	14	15	11	
Age * (years)	55.6 ± 3.3	56.9 ± 3.1	63.5 ± 1.6	*0.09*
Gender (F/M)	12/2	14/1	9/2	*0.67*
Smoker (Yes/No)	5/9	8/7	6/5	*0.55*
FT4 (pmol/L) *	11.9 ± 1.0	11.7 ± 1.0	9.8 ± 1.4	*0.15*
TRAb (mU/mL) *	27.6 ± 13.8	12.7 ± 3.0	11.6 ± 4.1	*0.93*
GO duration (months) *	4.9 ± 1.6	6.3 ± 1.5	10.7 ± 3.0	*0.14*
New onset/relapse (%)	4/10 (30)	9/6 (60)	7/4 (64)	*0.14*
CAS *	4.6 ± 0.3	4.3 ± 0.2	4.4 ± 0.3	*0.73*
Proptosis right eye (mm) *	23.8 ± 0.7	23.0 ± 0.6	21.4 ± 0.9	*0.13*
Proptosis left eye (mm) *	23.6 ± 1.0	22.6 ± 0.7	21.3 ± 0.9	*0.21*
Gorman score N (0/1/2/3)	4/4/2/4	3/4/7/1	4/2/4/1	*0.45*
CD19^+^ cells * (cells/mm^3^)	293.5 ± 24.7	236.5 ± 31.2	269.7 ± 50.4	*0.21*
GO-QoL Appearance * (%)	56.6 ± 6.3	61.0 ± 6.7	69.0 ± 13.0	*0.69*
GO-QoL Function *(%)	56.4 ± 9.7	42.3 ± 7.3	77.0 ± 14.6	*0.18*
Thyroid status (0 = Euthyroid, 1 = Hyperthyroid, 2 = Hypothyroid)	11/1/2	12/2/1	5/1/5	*0.15*
Thyroid diagnosis N (GD/HT/EGO)	11/3/0	12/2/1	11/0/0	0.66**
Thyroid disease duration	33.9 ± 9.3	44.1 ± 14.9	50.8 ± 35.6	*0.68*
N of GD patients treated with RAI/TX before RTX (on L-T4)	4 (3)	2 (2)	2 (1)	*0.58*

*All values are expressed as mean ± SE.

**in the Chi-Square analysis HT and EGO were added together.

CAS, clinical activity score; EGO, euthyroid Graves’ orbitopathy; GD, Graves’ disease; GO, Graves’ orbitopathy; HT, Hashimoto’ thyroiditis; L-T4, levothyroxine; QOL, quality of life; RAI, radioiodine treatment; RTX, rituximab; TX, total thyroidectomy.

Patients were defined as euthyroid (0) hyperthyroid (1) or hypothyroid (2) in case of normal, elevated or low FT3 and/or FT4, independently on TSH serum levels.

### RTX Administration Schedule

RTX dosing and treatment schedule were adapted based on the experience achieved with this drug throughout the years. The dose of RTX initially employed in the treatment of active moderate-to-severe GO was based on previous work in autoimmunity (6, 16, 17) and consisted of a single dose of 100 mg and 500 mg (Group 1 and Group 2, respectively) or 1000 mg twice at two weeks interval (Group 3).

All patients, one hour prior to RTX infusion, received oral paracetamol (1 gr), chlorphenamine (10 mg) and i.v. hydrocortisone (100 mg) to prevent possible infusion reactions.

Patients with severe chronic diseases, ongoing infections or neoplastic diseases were excluded from treatment. RTX was also not administered in patents with known significant coronary artery disease, cardiac arrhythmias, congestive heart failure, active infection, primary or secondary immunodeficiency, history of hypersensitivity, known anaphylaxis to mouse-derived proteins, positive Purified Protein Derivative (PPD) test without documentation of treatment for tuberculosis (TB) infection and denied consent to HIV testing. During treatment and follow-up, complete blood count, serum glucose, aminotransferases and gamma glutamyltransferase were closely monitored. Side effects were classified as major (diabetes mellitus, depressive syndrome, increase of aminotransferases levels 5 times or above the upper normal limit) and minor (dyspepsia, laryngeal itching, rhinorrhea, insomnia).

### Biochemical Analysis

Serum free-thyroxine (FT4), free-triiodothyronine (FT3) and TSH concentrations were measured using an electro-chemiluminescent immunoassay (ECLIA, Roche Diagnostics) and normal ranges were 8-17 pg/ml, 2-5 pg/ml and 0.26-5.2 mU/L, respectively. Serum TRAb were measured as TSH binding inhibitory immunoglobulins (TBII), using a 2nd generation TRAK human lumitest (Thermofisher, AG, Henningsdorf/Berlin, Germany; n.v.<1.5 U/L). Lymphocyte subpopulations were measured at baseline and at each follow-up examination.

### Cytofluorimetric Analysis

The pattern of peripheral blood lymphocytes was studied before RTX and subsequently during the study period and the follow-up. We tested the standard immunophenotypic panel (CD3+, CD3+4+, CD3+8+, CD3+DR+, CD20+, CD19+5+, CD56+16+3) on aliquots of around 10^5^ lymphocytes, submitted to triple staining procedures for immunogating with CD45, and the pairs of monoclonal antibodies to subpopulations of T, B and NK cells, subsequently processed in the flow cytometer (BD Facsan, Cell-quest software).

### Study Endpoints and Clinical Assessment

Primary endpoint of the study was GO inactivation, defined as a reduction of 2 points of CAS, or a CAS of 3/10 or less, at 12 and 24 weeks after treatment with any RTX dose, when compared to baseline. The analysis of efficacy was also carried out after stratifying patients for disease duration of more or less of six months at the time of intervention.

Secondary endpoints at 24 weeks were: 1) changes of the severity of GO: significant improvement was considered a 2 mm reduction of proptosis, and of at least one class of the Gorman’s score for diplopia (18); 2) improvement of the quality of life (QoL), assessed with a specific and validated questionnaire for GO (GO-QoL) (19): an increase of at least 6 points of the score of the questionnaire at 24 weeks was considered significant; 3) decrease of serum levels of TRAb; 4) decrease of peripheral B-cell lymphocytes; 5) occurrence of any major adverse events.

### Statistical Analysis

This was a *post-hoc* analysis of three prospective trials, one randomized to intravenous steroids and two open label. All values are expressed as mean ± standard error (SE) or ± standard deviation (SD), as specified. Analysis by Fischer exact test, Wilcoxon and Mann-Whitney test were applied, as appropriate, and performed using SPSS 8.0 for Windows. Statistical significance was defined as P <0.05. An ANOVA or Friedman model was used to study the changes of the CAS values during treatment and follow-up. A *per protocol* analysis of the data was carried out and patients undergoing surgical orbital decompression during the observation period up to 24 weeks, were not included in the analysis of the disease outcomes.

## Results

### Disease Inactivation and Relapse

There were no significant differences in baseline clinical and immunological features in the patients of the three groups of the study (**Table 1**).

In Group 1, baseline CAS value was 4.6 ± 0.3 and decreased to 2.1 ± 0.4 at 12 weeks (P<0.05) and to 1.1 ± 0.2 at 24 weeks (P<0.0001). In Group 2, baseline CAS was 4.3 ± 0.2 and decreased to 1.4 ± 0.4 (P<0.001) and to 0.5 ± 0.3 (P<0.0001) at 12 and 24 weeks, respectively. In Group 3, baseline CAS was 4.4 ± 0.3, 1.8 ± 0.4 (P<0.05) and 0.7 ± 0.2 (P<0.0001) at 12 and 24 weeks, respectively (**Table 2**).

**Table 2:** Clinical activity score (CAS) at baseline and 12 and 24 weeks after treatment with different doses of RTX.

	Baseline	12 weeks	P*	24 weeks	P*0 vs 24 weeks	*P* *12 vs 24 weeks
**GROUP 1** **100 mg**	4.6 ± 0.3	2.1 ± 0.4	*<0.05*	1.1 ± 0.2	*<0.0001*	*NS*
**GROUP 2** **500 mg**	4.3 ± 0.2	1.4 ± 0.4	*<0.001*	0.5 ± 0.3	*<0.0001*	*NS*
**GROUP 3** **1000 mg X2**	4.4 ± 0.3	1.8 ± 0.4	*<0.05*	0.7 ± 0.2	*<0.0001*	*NS*

*Friedman TEST.

All values are expressed as mean ± SE. NS, not significant.

**Figure 1:**
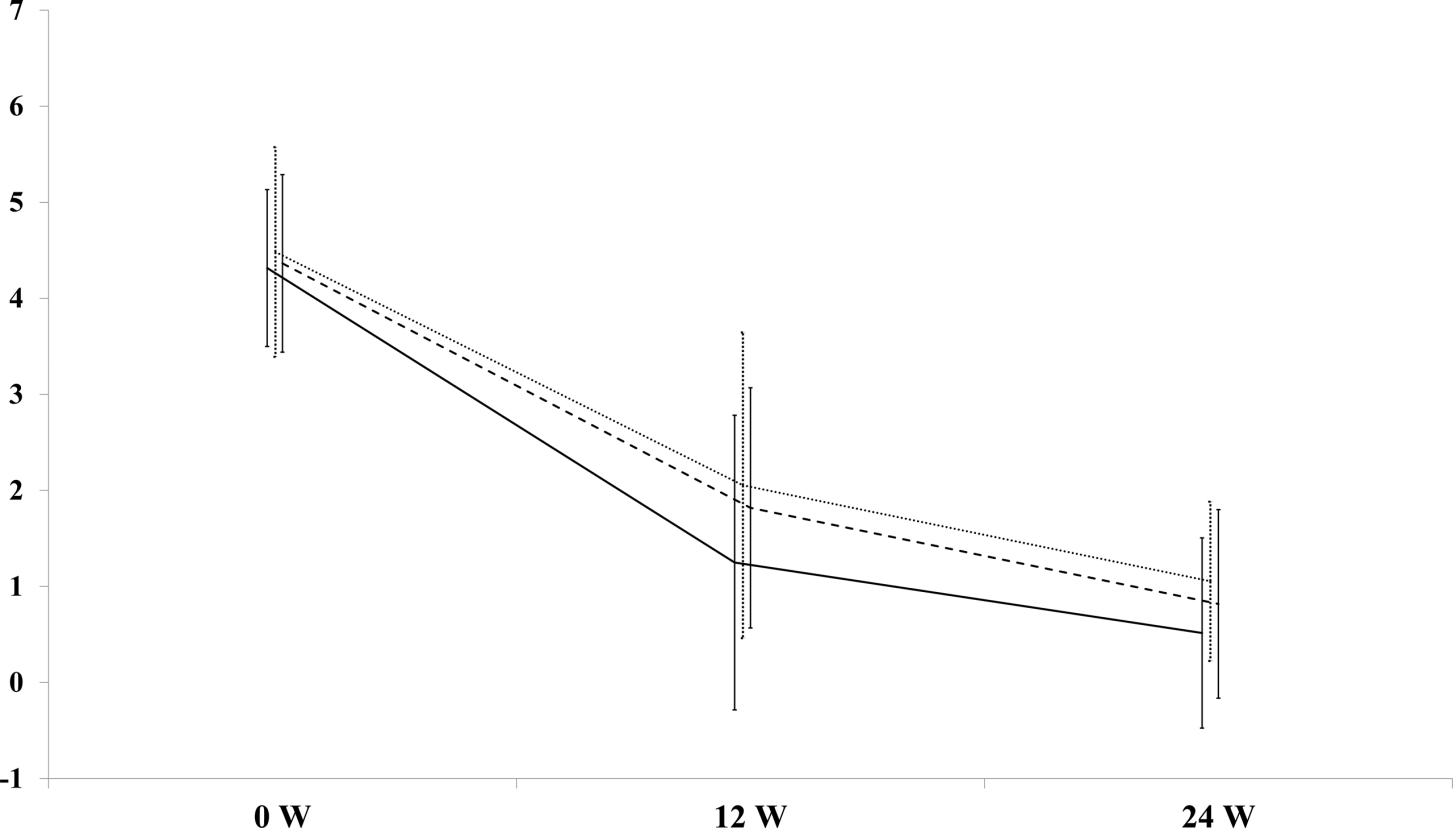
Decrease of the Clinical Activity Score (CAS) at 12 and 24 weeks after rituximab (RTX) in patients with Graves’ orbitopathy (GO). Dotted line= Group 1 (RTX 100 mg); solid line=Group 2 (RTX 500 mg); long dash line=Group 3 (RTX 1000 mg x 2).

There was no difference in the CAS reduction at 12 weeks in the three Groups of patients (2.6 ± 0.4 in Group 1, 2.9 ± 0.4 in Group 2, 2.6 ± 0.4 points in Group 3; P=N.S). No difference was also observed at 24 weeks (3.6 ± 0.3 in Group 1, 3.8 ± 0.3 in Group 2 and 3.6 ± 0.4 in Group 3; P=N.S; **Figure 1**). GO inactivation was not significantly associated to disease duration (more or less than 6 months) independently of the dose employed (not shown, P=N.S). Response to RTX therapy was not different whether patients had newly occurring GO or relapse after previous steroid therapy (not shown, P=N.S.).

No patient showed disease reactivation at 24 weeks and during follow-up to 76 weeks. Two patients treated with 100 mg RTX, despite an initial response to treatment, eventually developed dysthyroid optic neuropathy (DON) and required prompt surgical orbital decompression.

### Disease Severity and Quality of Life

Mean proptosis values did not significantly change at 24 weeks in any of the 3 groups after RTX (**Table 3**).

**Table 3:** Clinical outcome of patients with moderate severe GO assessed 24 weeks after therapy with different doses of RTX.

	GROUP 1 (100 mg)	GROUP 2 (500 mg)	GROUP 3 (1000 mg x2)	P*
Number of patient	14	15	11	
TRAb (mU/L)	14.9 ± 7.5	5.98 ± 1.98	10.5 ± 4.0	*0.48*
CAS	1.07 ± 0.2	0.5 ± 0.3	0.7 ± 0.2	*0.14*
Proptosis right eye (mm)	22.6 ± 0.8	22.97 ± 0.7	22.05 ± 1.4	*0.73*
Proptosis left eye (mm)	23.0 ± 0.9	22.7 ± 0.6	21.5 ± 1.1	*0.58*
Δ Proptosis right eye (mm)**	-1.2 ± 0.4	-0.08 ± 0.3	0.64 ± 1.1	*0.08*
Δ Proptosis left eye (mm)**	-0.6 ± 0.6	0.10 ± 0.2	0.2 ± 0.5	*0.50*
GO-QoL Appearance (%)	71.6 ± 4.6	59.8 ± 6.4	83.2 ± 5.97	*0.18*
GO-QoL Functions (%)	52.0 ± 9.8	58.9 ± 9.2	72.3 ± 16.8	*0.66*
CD19^+^ cells (cells/mm^3^)	75.1 ± 15.3	27.1 ± 7.1	7.5 ± 4.3	*G1* vs *G2 P<0.05; G1* vs *G3 P<0.0001; G2* vs *G3 P = NS*

All values are expressed as mean ± SE

*Mann-Whitney test

**Difference in between proptosis at 0 and 24 weeks.

GO, Graves’ orbitopathy; CAS, clinical activity score; GO-QoL, quality of life questionnaire; NS, not significant.

**Figure 2:**
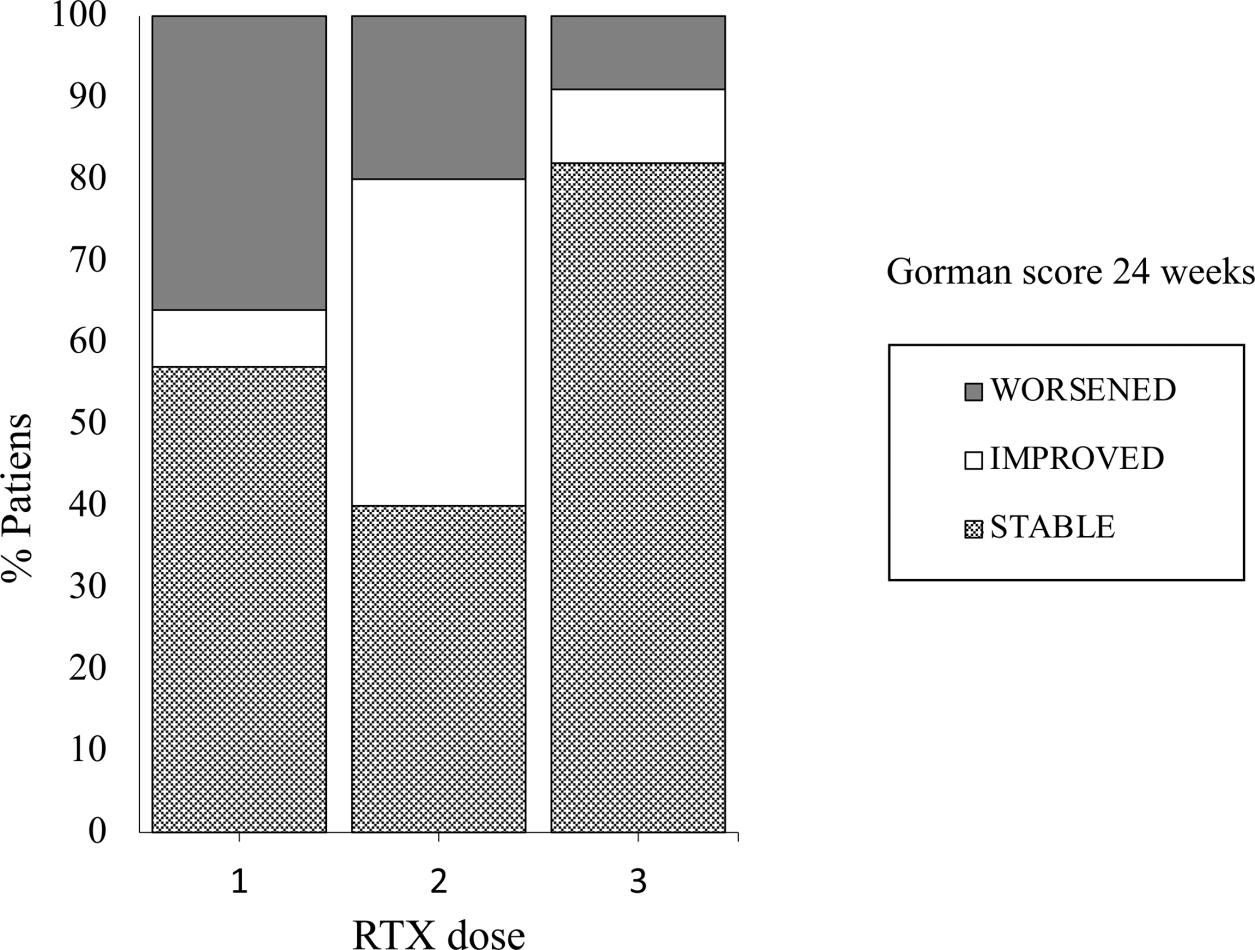
Proportions of patients with GO with modifications of the Gorman score for diplopia after treatment with three different doses of RTX: 1= Group 1 (RTX 100 mg); 2= Group 2 (RTX 500 mg); 3 Group 3 (RTX 1000 mg x 2). Chi Square test.

Patients receiving 500 mg of RTX tended to have more improved or stable diplopia (Chi-square P<0.06; **Figure 2**), and this finding was confirmed by the observation of a significant inverse correlation of the Gorman score with the dose of RTX (r -0.43, P< 0.01).

**Figure 3:**
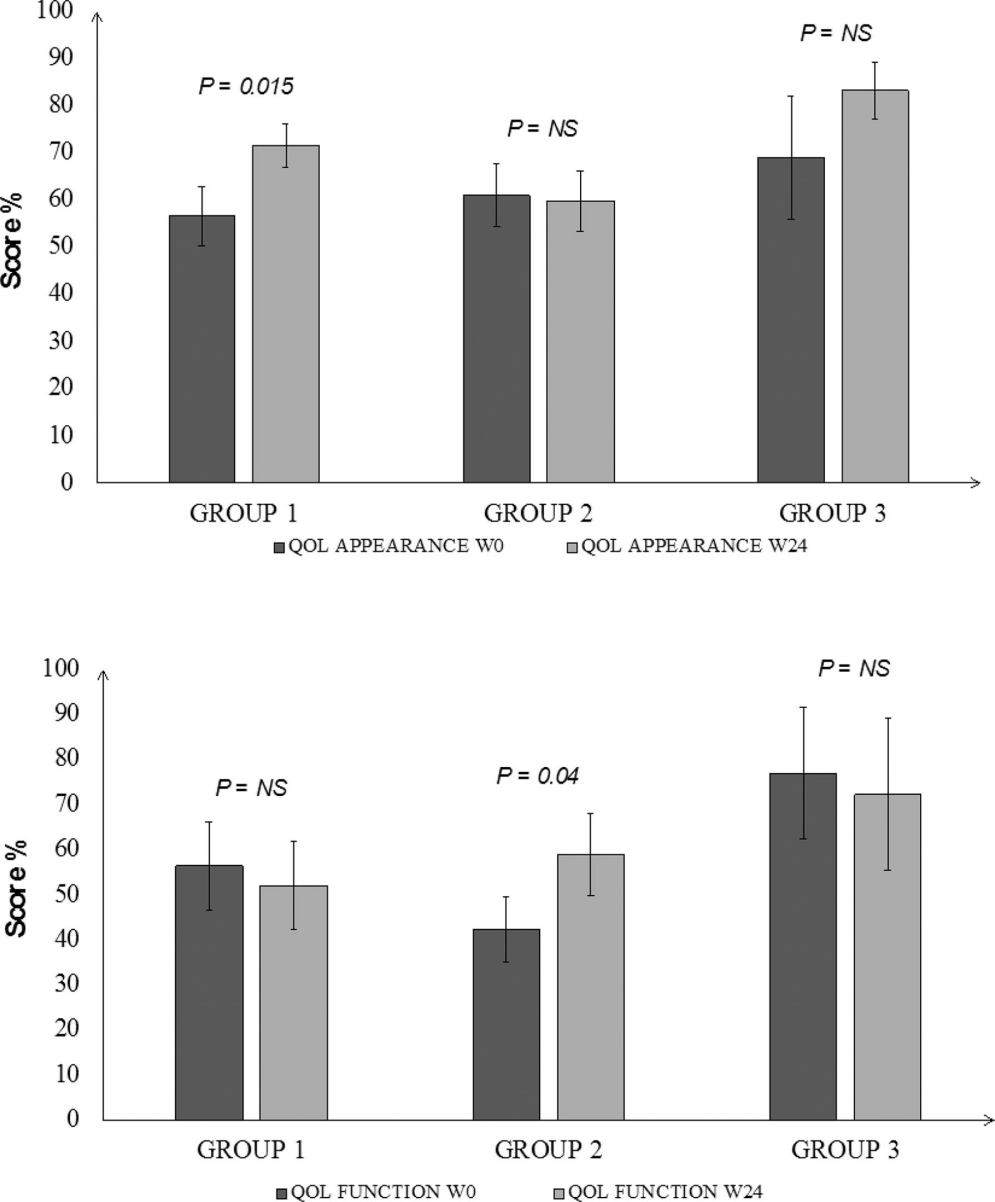
Changes in the baseline score of the Quality of Life assessment of patients with GO at 24 weeks after treatment with different doses of RTX. Panel **(A)** (upper): appearance score, Panel **(B)** (lower): function score. Group 1 = 100 mg; Group 2 = 500 mg; group 3 = 1000 mg x 2; baseline values are in dark grey and values at 24 weeks in light grey. Wilcoxon matched pair test.

In Group 1 the appearance score of the GO-QoL questionnaire improved in 6/14 patients and the visual function score in 4/14 (**Table 3**), with significant increase of the overall appearance score (P=0.015, **Figure 3A**), but not of the function score (**Figure 3B**). In Group 2 improvement of the appearance score was observed in 7/15 patients and in one of the visual function score, although improvement was significant only for the overall visual function score (P=0.04, **Figure 3B**), and not of the appearance score (**Figure 3A**). In Group 3 analysis of the overall score was not performed due to the low number of patients who completed the questionnaire.

### Thyroid Function and Serum TRAb

**Figure 4:**
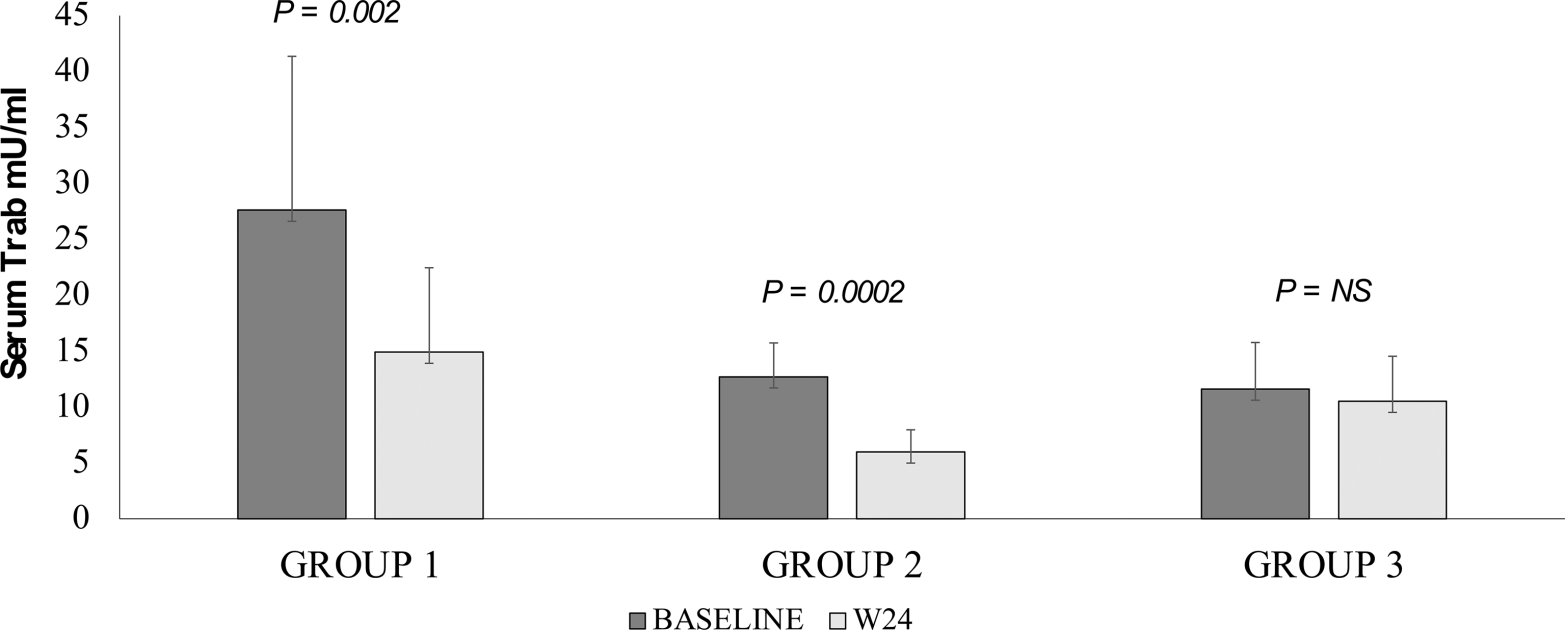
Changes of serum TRH receptor antibody levels at 24 weeks in patients with GO treated with different doses of RTX. Group 1 = 100 mg; Group 2 = 500 mg; group 3 = 1000 mg x 2. Baseline values are in dark grey and values at 24 weeks in light grey. Paired T test.

All but one patients at 24 weeks were euthyroid (P=0.04, not shown). Detailed data on baseline treatments for the thyroid diseases are reported in **Table 1**. A significant reduction of serum TRAb levels at 24 weeks was observed after RTX in Group 1 (P=0.002) and Group 2 (P<0.0002), but not in Group 3 patients (P=NS; **Figure 4**).

### Lymphocytes Depletion

**Figure 5:**
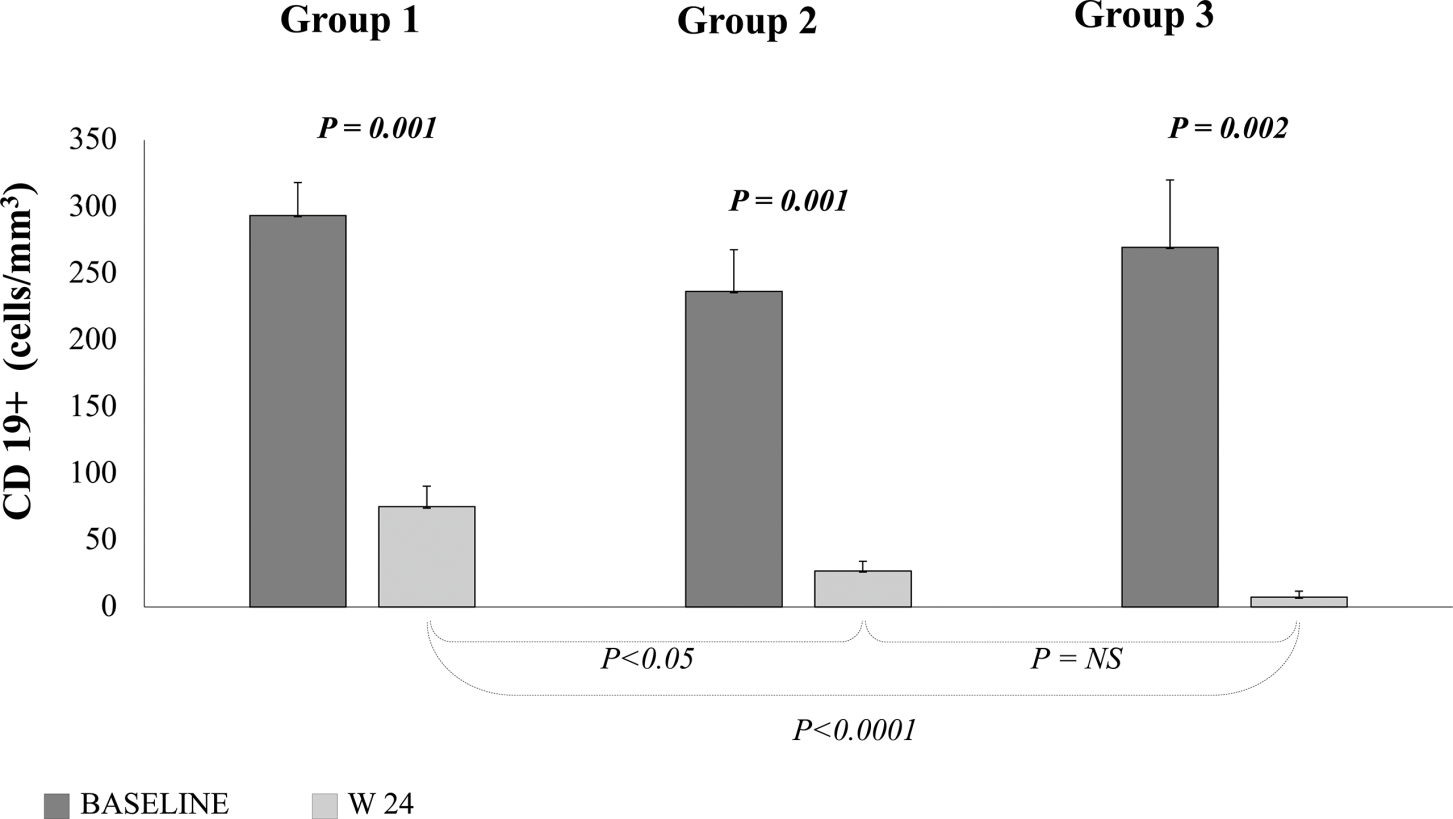
Peripheral B cell count measured at baseline (dark grey) and 24 weeks (light grey) after treatment with different doses of RTX. Group 1 = 100 mg; Group 2 = 500 mg; Group 3 = 1000 mg x 2. Paired T test.

Peripheral lymphocyte subpopulations were studied at 24 weeks in order to assess how different RTX doses affect CD19+ cell depletion. A significant reduction in CD19 cell numbers was observed in all three groups (P< 0.01), independently of the RTX dose employed (**Figure 5**). At 24 weeks, the number of CD 19+ cells in patients of Group 1 were 75.1 ± 15.3, significantly higher than in patients of Group 2 (27.1 ± 7.1; P=0.05) Group 3 (7.5 ± 4.3; P=0.0001), while the difference is not statistically significant between patients of Group 2 and Group 3 (**Table 3**).

Overall, rehabilitative surgery (evaluated at 76 weeks post-treatment with RTX) was performed in 20 patients. Ten patients underwent orbital decompressions, of whom eight elective and two emergency (DON), six patients strabismus surgery and four eyelid surgery. The distribution of surgical procedures among patients was not related to the RTX dose employed.

We did not observe any significant changes in the CD4+ or CD8+ T lymphocytes levels following RTX infusion, in any Groups (**Supplementary Figure 1**). Interestingly, we observed a slightly increase in the number of natural killer cells (NK) in Group 1 and 2 after RTX. This difference reached the statistical significance only at 8 weeks in Group 1 and at 32 and 40 weeks in Group 2 compared to baseline values. On the contrary, the number of NK cells remained stable in Group 3 (**Supplementary Figure 1**).

### Adverse Events

Minor adverse reactions such as itching of the throats and stuffiness of nose with rhinorrhea, were experienced by many patients. In these cases, resolution of the symptoms occurred after slowing down the rate of RTX infusion. In two patients of Group 1 a major adverse event known as the syndrome of release of cytokines occurred early during RTX infusion. This was characterized by orbital edema and progressive decline of vision rapidly occurring 30 minutes after the beginning of the infusion. This reaction was controlled by the administration of 100 mg of i.v. hydrocortisone with complete recovery of vision after 3 hours (9). Two patients treated with 100 mg RTX developed DON, possibly present at a subclinical stage at the time of treatment. This event is to be considered treatment failure, rather than an adverse event.

### Cost of Treatment

The cost of RTX therapy depends the dose employed. The actual cost of one single 100 mg dose is of € 338, that of one 500 mg dose is € 1,698 and that of a two-dose cycle of 1000 mg is € 2.154, as released by the manufacturer in Europe (Roche Ltd.)

## Discussion

The first and most important finding of this study is that doses of RTX ranging from 100 to 2000 mg invariably induce inactivation of moderate-to-severe GO with only some differences in the clinical response, especially on diplopia, quality of life assessment and CD 19+ cells repopulation. This study is based on a *post-hoc* analysis of data collected from one prospective RCT and two open label studies that have employed different protocols and dosing of RTX administration.

Our findings show that any of the three RTX dosages used (100 mg, 500 mg and 1000 mg x 2) is equally effective in inactivating the disease in >95% of patients at 12 and 24 weeks after infusion. Efficacy of RTX has been known to be associated to the duration of B cell depletion in the circulation (14). In this study we show that peripheral CD19+ cells begin repopulating only slightly earlier than 24 weeks in patients treated with the very low dose (100 mg), but later in those administered either 500 or 2000 mg. B cell return in the circulation, though, does not modify the disease outcome or favor disease relapse during follow-up (up to 12 months) (6, 14, 20). Based on these observations, we believe that in moderate-severe GO lower doses of RTX, which are generally burdened by a lower risk of adverse events such as reactivation of infections (12) or the induction of other autoimmune processes after depletion of B cells (21), should be preferred.

We studied whether RTX induced inactivation may depend on GO duration of less or more than 6 months, as previous studies have suggested that in GO of longer duration RTX might be less effective (7, 17). We did not find differences in the therapeutic response based on the duration of disease with any of the doses employed. We suggest that efficacy of RTX treatment is likely more related to the early inflammatory phase of GO, than to disease duration. We observed no difference in the response of patients with newly diagnosed GO or of those with relapsing disease after a complete cycle of intravenous steroids, who invariably had longer disease duration (up to 66 months). This findings, besides confirming that disease activity rather than duration is important for the effect of RTX in GO, also suggest that this modality of therapy can be proposed as second line treatment when steroids fail (1, 2).

The therapeutic outcome on parameters of disease severity, especially those related to muscle involvement, appear to be of importance, as the presence of diplopia in inactive GO has an impact on the quality of life of patients. While there were no significant changes in the degree of proptosis with any of the three dosages used, we did observe a significant improvement of the Gorman score for diplopia when higher RTX doses (Group 2 and Group 3) were employed. A possible explanation for this is that higher doses might be more effective than lower doses in preventing the orbital tissue fibrosis that follows the active disease phase.

The QoL questionnaire used in the study has been validated and is specific for assessing quality of life changes in GO patients (18). It is divided into two sections, the first one assessing the impact of the aesthetic changes (appearance) on patients, the second one that of the eye dysfunction. The perception of self-appearance was found to improve at 24 weeks only in patients treated with 100 mg RTX in a single administration, while improvement of function was reported by those treated with a single larger dose of 500 mg. Improvement of inflammatory signs were in fact more rapidly observed after treatment with the low dose (>90% of patients inactivated at 12 weeks), whereas the impact of RTX on diplopia was found to be associated to the use of larger doses. This suggests that high doses of RTX might be preferable in patients with significant extraocular muscle involvement, as it would reduce the need of rehabilitative surgery. Unfortunately, we could not assess QoL outcome in Group 3 because of the small number of questionnaires returned, and this is a limitation of this study. Differences in the quality of life assessment were not associated to differences in the number of rehabilitative surgeries required, whichever dose of RTX was employed.

In two patients treated with the low dose, RTX therapy failed to inactivate GO and to prevent progression to DON. These patients may have had a subclinical form of DON at the time of treatment which might have been further exacerbated by the increase of intraorbital edema induced by an acute release of cytokines mediated by RTX as previously reported (9). In other studies RTX has been used in patients with DON (10) resulting in improvement of vision. In addition, the observation of the syndrome cytokine release in two patients receiving only 100 mg RTX, suggests that this adverse event is not related to the dose employed and can be prevented only by pretreating patients with fairly high intravenous methylprednisolone doses (up to 500 mg) (22). Caution is therefore suggested for the use of RTX in patients with suspected subclinical DON (1).

Restoration of euthyroidism after RTX may be due to either its effect on antibodies stimulating the TSH receptor (23) or to remission of hyperthyroidism after prolonged antithyroid treatment (24). While some authors have hypothesized that RTX does induce a decrease of serum TRAb in patients with GD (25, 26), others have shown that the levels of antibodies stimulating the TSH receptor (TSAb) are not modified in GO patients (23). RTX causes depletion of B lymphocytes in the peripheral blood but also in the thyroid (27) and in the orbit (9) and histological analysis of orbital tissues of patients who received RTX showed absence of both B and T lymphocytes (8). This observation supports the hypothesis that RTX blocks B cell function as antigen presenting cells and interaction with helper T cells, required for the initiation of the autoimmune process (28). Interestingly, we observed that the number of NK cells increased following RTX infusion in Group 1 and 2, compared to baseline values. Previous studies on rheumatoid arthritis and idiopathic membranous nephropathy have shown a rise in the NK number, associated with an altered NK cell function. Although, the clinical significance of this observation needs to be established, it has been associated with a better response to RTX treatment (29, 30).

This may explain why after CD19+ cells repopulation, inflammation does not relapse and the CAS continues to decrease until stable disease inactivation (31). We in fact did observe that although CD19+ cells return earlier (<24 weeks) in patients treated with the 100 mg dose, when compared to Groups 2 and 3, this does not result in GO reactivation.

In analyzing the use of RTX in GO, we also looked at the cost of therapy in relation to the administered dose. When compared to first line treatment with intravenous steroids, the cost of a full cycle of treatment with RTX in Europe is about 4.8, 24 and 61 times more expensive for the 100, 500 and 1000 x 2 mg dose, respectively. Given the more favorable response/relapse rate and the clinical evidence shown by this study, we believe that a single RTX 500 mg dose is best indicated as second line treatment for the majority of GO patients.

The results of this study have to be seen in light of some limitations: in first instance the retrospective study design, the small sample size and its heterogeneity. Indeed, cases had a different duration of GO (which was longer in patients belonging to Group 3 compared to Group 1 and 2) and were enrolled among Graves’disease, Hashimoto’s thyroiditis and euthyroid GO patients. Furthermore, some patients received previous corticosteroid treatment that may have had influenced the final outcome.

In this study, in conclusion, we sought to understand, from the limited available evidence, which dose of RTX is optimal as second line therapy of active moderate-to-severe GO. In this analysis we considered, both the effectiveness of the drug in inactivating the disease, the possibility of changing its natural course, the effect on disease severity and the impact on the patients’ quality of life. Prospective randomized studies specifically addressing RTX dosing in GO are warranted.

## Data Availability Statement

The datasets presented in this article are not readily available because they contain some sensitive personal data. Requests to access the datasets should be directed to the corresponding author.

## Ethics Statement

The studies involving human participants were reviewed and approved by Fondazione IRCCS Ca’ Granda, Ospedale Policlinico, Milan, Italy. The patients/participants provided their written informed consent to participate in this study.

## Author Contributions

Data of each trial were collected by IC, DC, GV, MS, EL, LF, NC, LP and CG, BM and MD performed some serological assays and collected biochemical and cytofluorimetric data, the final database was produced by IM and EL, results were analyzed by IC and EL, the draft of the manuscript was written by IM and MS, the final manuscript was reviewed and revised by MS and MA. All authors contributed to the article and approved the submitted version.

## Funding

This work was supported by funds of Fondazione Ca’ Granda, IRCCS, Milano, Italy to MS (RC 2020).

## Conflict of Interest

The authors declare that the research was conducted in the absence of any commercial or financial relationships that could be construed as a potential conflict of interest.

## Publisher’s Note

All claims expressed in this article are solely those of the authors and do not necessarily represent those of their affiliated organizations, or those of the publisher, the editors and the reviewers. Any product that may be evaluated in this article, or claim that may be made by its manufacturer, is not guaranteed or endorsed by the publisher.

## Supplementary Material

Supplementary Figure 1 Peripheral T cell, CD4+ (light gray) and CD8+ (white) and NK (dark grey) count measured at baseline and at different time-points after treatment with different doses of RTX. Test: Repeated measure ANOVA.

## References

[1] Bartalena L Baldeschi L Boboridis K Eckstein A Kahaly GJ Marcocci C. The 2016 European Thyroid Association/European Group on Graves’ Orbitopathy Guidelines for the Management of Graves’ Orbitopathy. Eur Thyroid J (2016) 5:9–26. doi: 10.1159/000443828 27099835 PMC4836120

[2] Bartalena L Kahaly GJ Baldeschi L Dayan CM Eckstein A Marcocci C. EUGOGO †. The 2021 European Group on Graves’ Orbitopathy (EUGOGO) Clinical Practice Guidelines for the Medical Management of Graves’ Orbitopathy. Eur J Endocrinol (2021) 185:G43–67. doi: 10.1530/EJE-21-0479 34297684

[3] Bartley GB. Rundle and His Curve. Arch Ophthalmol (2011) 129:356–8. doi: 10.1001/archophthalmol.2011.29 21402995

[4] Bartalena L Krassas GE Wiersinga W Marcocci C Salvi M Daumerie C. Efficacy and Safety of Three Different Cumulative Doses of Intravenous Methylprednisolone for Moderate to Severe and Active Graves’ Orbitopathy. J Clin Endocrinol Metab (2012) 97:4454–63. doi: 10.1210/jc.2012-2389 23038682

[5] Dorner T Lipsky PE. Beyond Pan-B-Cell-Directed Therapy – New Avenues and Insights Into the Pathogenesis of SLE. Nat Rev Rheumatol (2016) 12:645–57. doi: 10.1038/nrrheum.2016.158 27733759

[6] Salvi M Vannucchi G Currò N Campi I Covelli D Dazzi D. Efficacy of B-Cell Targeted Therapy With Rituximab in Patients With Active Moderate to Severe Graves’ Orbitopathy: A Randomized Controlled Study. J Clin Endocrinol Metab (2015) 100:422–3. doi: 10.1210/jc.2014-3014 PMC4318899 25494967

[7] Stan MN Salvi M. Management of Endocrine Disease: Rituximab Therapy for Graves’ Orbitopathy - Lessons From Randomized Control Trials. Eur J Endocrinol (2017) 176:R101–109. doi: 10.1530/EJE-16-0552 27760790

[8] Salvi M Vannucchi G Campi I Rossi S Bonara P Sbrozzi F. Efficacy of Rituximab Treatment for Thyroid-Associated Ophthalmopathy as a Result of Intraorbital B-Cell Depletion in One Patient Unresponsive to Steroid Immunosuppression. Eur J Endocrinol (2006) 154:511–7. doi: 10.1530/eje.1.02119 16556712

[9] Salvi M Vannucchi G Currò N Introna M Rossi S Bonara P. Small Dose of Rituximab for Graves’ Orbitopathy: New Insights Into the Mechanism of Action. Arch Ophthalmol (2012) 130:122–4. doi: 10.1001/archopthalmol.2011.1215 22232486

[10] Khanna D Chong KK Afifiyan NF Hwang CJ Lee DK Garneau HC. Rituximab Treatment of Patients With Severe, Corticosteroid-Resistant Thyroid-Associated Ophthalmopathy. Ophthalmology (2010) 117:133–9. doi: 10.1016/j.ophtha.2009.05.029 PMC2814962 19818507

[11] Descotes J. Immunotoxicity of Monoclonal Antibodies. MAbs (2009) 1:104–11. doi: 10.4161/mabs.1.2.7909 PMC2725414 20061816

[12] van Vollenhoven RF Emery P Bingham CO III Keystone EC Fleischmann RM Furst DE. Long-Term Safety of Rituximab in Rheumatoid Arthritis: 9.5- Year Follow-Up of the Global Clinical Trial Programme With a Focus on Adverse Events of Interest in RA Patients. Ann Rheum Dis (2013) 72:1496–502. doi: 10.1136/annrheumdis-2012-201956 PMC3756452 23136242

[13] Winkler U Jensen M Manzke O Schulz H Diehl V Engert A. Cytokine-Release Syndrome in Patients With B-Cell Chronic Lymphocytic Leukemia and High Lymphocyte Counts After Treatment With an Anti-CD20 Monoclonal Antibody (Rituximab, IDEC-C2b8). Blood (1999) 94:2217–24. doi: 10.1182/blood.V94.7.2217.419k02_2217_2224 10498591

[14] Vannucchi G Campi I Covelli D Currò N Lazzaroni E Palomba A. Efficacy Profile and Safety of Very Low-Dose Rituximab in Patients With Graves’ Orbitopathy. Thyroid (2021) 31:821–8. doi: 10.1089/thy.2020.0269 33234032

[15] Salvi M Vannucchi G Campi I Currò N Dazzi D Simonetta S. Treatment of Graves’ Disease and Associated Ophthalmopathy With the Anti-CD20 Monoclonal Antibody Rituximab: An Open Study. Eur J Endocrinol (2007) 156:33–40. doi: 10.1530/eje.1.02325 17218723

[16] Bredemeier M Campos GG de Oliveira FK. Updated Systematic Review and Meta-Analysis of Randomized Controlled Trials Comparing Low- Versus High-Dose Rituximab for Rheumatoid Arthritis. Clin Rheumatol (2015) 34:1801–5. doi: 10.1007/s10067-015-2977-z 26070536

[17] Stan MN Garrity JA Carranza Leon BG Prabin T Bradley EA Bahn RS. Randomized Controlled Trial of Rituximab in Patients With Graves’ Orbitopathy. J Clin Endocrinol Metab (2015) 100:432–41. doi: 10.1210/jc.2014-2572 PMC4318907 25343233

[18] Bahn RS Gorman CA. Choice of Therapy and Criteria for Assessing Treatment Outcome in Thyroid-Associated Ophthalmopathy. Endocrinol Metab Clin North Am (1987) 16:391–407. doi: 10.1016/S0889-8529(18)30485-7 3319588

[19] Terwee CB Gerding MN Dekker FW Prummel MF Wiersinga WM. Development of a Disease Specific Quality of Life Questionnaire for Patients With Graves’ Ophthalmopathy: The GO-QOL. Br J Ophthalmol (1998) 82:773–9. doi: 10.1136/bjo.82.7.773 PMC1722683 9924370

[20] Erdei A Paragh G Kovacs P Karanyi Z Berenyi E Galuska L. Rapid Response to and Long-Term Effectiveness of Anti-CD20 Antibody in Conventional Therapy Resistant Graves’ Orbitopathy: A Five-Year Follow-Up Study. Autoimmunity (2014) 47:548–53. doi: 10.3109/08916934.2014.939266 25039242

[21] El Fassi D Nielsen CH Kjeldsen J Clemmensen O Hegedüs L. Ulcerative Colitis Following B Lymphocyte Depletion With Rituximab in a Patient With Graves’ Disease. Gut (2008) 57:714–5. doi: 10.1136/gut.2007.138305 18408106

[22] Deltour JB d’Assigny Flamen M Ladsous M Giovansili L Cariou B Caron P. Efficacy of Rituximab in Patients With Graves’ Orbitopathy: A Retrospective Multicenter Nationwide Study. Graefes Arch Clin Exp Ophthalmol (2020) 258:2013–21. doi: 10.1007/s00417-020-04651-6 32405700

[23] El Fassi D Banga JP Gilbert JA Padoa C Hegedüs L Nielsen CH. Treatment of Graves’ Disease With Rituximab Specifically Reduces the Production of Thyroid Stimulating Autoantibodies. Clin Immuno (2009) 130:252–8. doi: 10.1016/j.clim.2008.09.007 18964302

[24] Vannucchi G Campi I Bonomi M Covelli D Dazzi D Currò N. Rituximab Treatment in Patients With Active Graves’ Orbitopathy: Effects on Proinflammatory and Humoral Immune Reactions. Clin Exp Immunol (2010) 161:436–43. doi: 10.1111/j.1365-2249.2010.04191.x PMC2962960 20529087

[25] El Fassi D Nielsen CH Bonnema SJ Hasselbalch HC Hegedüs L. B Lymphocyte Depletion With the Monoclonal Antibody Rituximab in Graves’ Disease: A Controlled Pilot Study. J Clin Endocrinol Metab (2007) 92:1769–72. doi: 10.1210/jc.2006-2388 17284622

[26] Mitchell AL Gan EH Morris M Johnson K Neoh C Dickinson AJ. The Effect of B Cell Depletion Therapy on Anti-TSH Receptor Antibodies and Clinical Outcome in Glucocorticoid-Refractory Graves’ Orbitopathy. Clin Endocrinol (2013) 79:437–42. doi: 10.1111/cen.12141 23320840

[27] El Fassi D Clemmensen O Nielsen CH Silkiss RZ Hegedüs L. Evidence of Intrathyroidal B-Lymphocyte Depletion After Rituximab Therapy in a Patient With Graves’ Disease. J Clin Endocrinol Metab (2007) 92:3762–3. doi: 10.1210/jc.2007-1238 17933978

[28] Salvi M Covelli D. B Cells in Graves’ Orbitopathy: More Than Just a Source of Antibodies? Eye (2019) 33:230–4. doi: 10.1038/s41433-018-0285-y PMC6367428 30514895

[29] Giollo A Viapiana O Carletto A Ortolani R Biasi D Gatti D. Rituximab Increases Peripheral Natural Killer Cells in Patients With Rheumatoid Arthritis. Clin Exp Rheumatol (2017) 35:241–6. 27908302

[30] Rosenzwajg M Languille E Debiec H Hygino J Dahan K Simon T. And T-Cell Subpopulations in Patients With Severe Idiopathic Membranous Nephropathy may Predict an Early Response to Rituximab. Kidney Int (2017) 92:227–37. doi: 10.1016/j.kint.2017.01.012 28318628

[31] Rotondo Dottore G Torregrossa L Caturegli P Ionni I Sframeli A Sabini E. Association of T and B Cells Infiltrating Orbital Tissues With Clinical Features of Graves Orbitopathy. JAMA Ophthalmol (2018) 136:613–9. doi: 10.1001/jamaophthalmol.2018.0806 PMC6583879 29710102

