# Supplementary figures and images for: Therapy With Different Dose Regimens of Rituximab in Patients With Active Moderate-To-Severe Graves’ Orbitopathy
Source: Front Endocrinol (Lausanne). 2022 Jan 25;12:790246. doi: 10.3389/fendo.2021.790246 (PMC8822584; doi:10.3389/fendo.2021.790246)

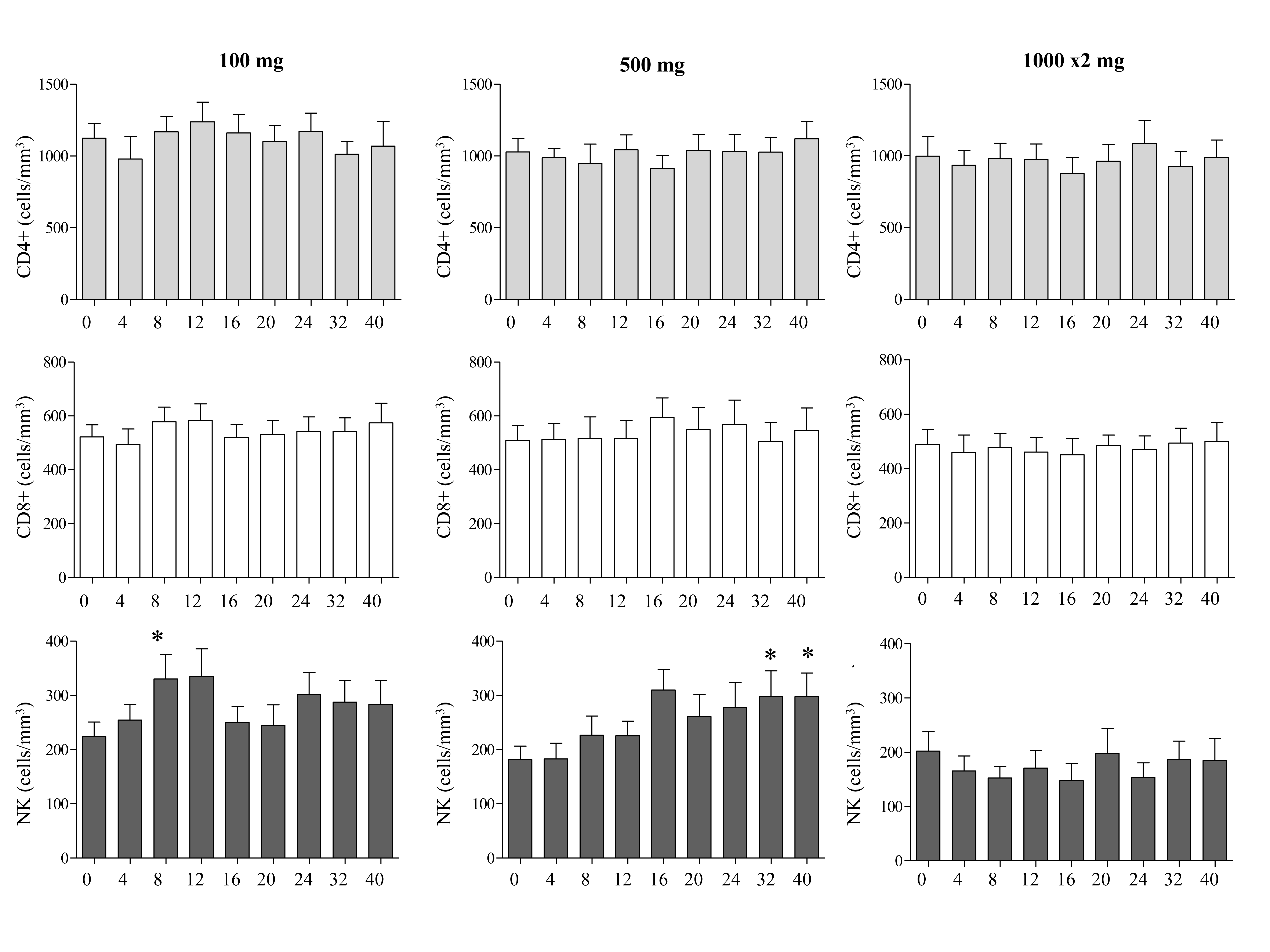

Supplement: Supplementary Figure 1 — Peripheral T cell, CD4+ (light gray) and CD8+ (white) and NK (dark grey) count measured at baseline and at different time-points after treatment with different doses of RTX. Test: Repeated measure ANOVA. [file Image_1.tif]
